# Supplementation of direct-fed microbial *Enterococcus faecium* 669 affects performance of preweaning dairy calves

**DOI:** 10.3168/jdsc.2022-0344

**Published:** 2023-04-18

**Authors:** Bruno I. Cappellozza, Giuseppe Copani, Erik J. Boll, Oscar Queiroz

**Affiliations:** Chr. Hansen A/S, Hørsholm, Denmark 2970

## Abstract

Optimization and support of health and performance of preweaning dairy calves is paramount to any dairy operation, and natural solutions, such as probiotics, may help to achieve such a goal. Two experiments were designed to evaluate the effects of direct-fed microbial (DFM) *Enterococcus faecium* 669 on performance of preweaning dairy calves. In experiment 1, twenty 4-d-old Holstein calves [initial body weight (BW) 41 ± 2.1 kg] were randomly assigned to either (1) no probiotic supplementation (CON; n = 10) or (2) supplementation with probiotic strain *E. faecium* 669 during the preweaning period (DFM; n = 10) at 2.0 × 10^10^ cfu/kg of whole milk. Full individual BW was analyzed every 20 d for average daily gain (ADG) and feed efficiency (FE) determination. In experiment 2, thirty 4-d-old Holstein calves (initial BW 40 ± 1.9 kg) were assigned to the same treatments as in experiment 1 (CON and DFM). The DFM supplementation period was divided into period I (from d 0 to 21) and II (from d 22 to 63), with weaning occurring when animals were 67 d of age. During the entire experimental period, DFM was mixed into the whole milk at a rate of 1.5 × 10^10^ and 2.5 × 10^9^ cfu/kg of whole milk/calf per day for periods I and II, respectively (6-time reduction). Full individual BW was taken every 21 d. As a routine of the experiment, calves were monitored daily, and diarrhea cases were evaluated using a daily 3-point fecal score. For both experiments, all data were analyzed using calf as the experimental unit. In experiment 1, DFM-supplemented calves were heavier on d 40 (+ 4.5 kg) and 60 (+ 6.5 kg) and had a greater ADG (+ 118 g) versus CON. In experiment 2, supplementation with DFM significantly tended to reduce diarrhea occurrence. Treatment × day and treatment × week interactions were observed for BW, ADG, and gain-to-feed ratio. Dairy calves supplemented with DFM were 1.8 and 3.5 kg heavier on d 42 and at weaning, respectively, and had a greater ADG from d 21 to 42 (+ 52 g) and 42 to 63 (+ 77 g) and gain-to-feed ratio from d 42 to 63 (+ 8.6%). In summary, supplementation of *E. faecium* 669 to dairy calves improved preweaning performance, even when the dose of the DFM was reduced by 6- to 8-times. Additionally, initial promising results were observed on diarrhea occurrence, but further studies are warranted.

## Highlights

• Probiotics, which are defined as live microorganisms benefiting the health of a host, might be a feasible tool to support an adequate performance of the herd.
• *Enterococcus faecium* 669 supplementation improved performance of preweaning dairy calves, even when different doses were fed.
• *Enterococcus faecium* 669 supplementation also reduced occurrence of diarrhea in experiment 2.

## Graphical Abstract

**Figure:**
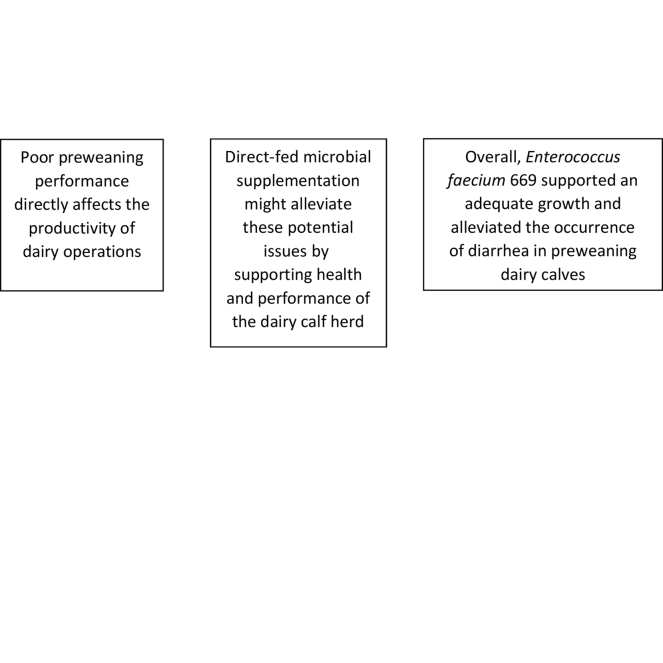
**Summary:** Optimization of preweaning performance might have medium- to long-term effects on the dairy cow herd. Hence, the use of technologies that promote preweaning performance is warranted and deserves to be investigated. One such technology could be direct-fed microbials, which support health and performance of ruminants. To investigate this, we fed *Enterococcus faecium* 669 to preweaning dairy Holstein calves. Overall, feeding *E. faecium* 669 improved average daily gain and yielded heavier calves at weaning, and a tendency for a reduced occurrence of diarrhea was also observed. In summary, feeding *E. faecium* 669 was a feasible strategy to promote performance of preweaning dairy calves.

The preweaning period has been identified as a critical timeframe of a calf's life that can affect short-, mid-, and long-term performance of the developing heifer and mature cow (Soberon et al., 2012; Bach et al., 2021). As an example, other researchers demonstrated that milk production is positively correlated with ADG from birth to breeding and that for every 1 kg of ADG during the preweaning period, 305-d milk yield increased up to 1,114 kg during the first lactation (Soberon et al., 2012). Hence, it is desirable to achieve a greater performance during the preweaning period to ensure an adequate productive performance in the subsequent lactations of the mature lactating dairy cow.

Probiotics are live microorganisms that, when offered in adequate amounts, provide health benefits to the host (FAO/WHO, 2001; Markowiak and Śliżewska, 2018). Among the probiotic strains, the lactic acid bacteria *Enterococcus faecium* 669 strain has been approved for use in calves up to 6 mo of age (EFSA, 2012) and is considered safe to use for the animal and for the human population, as no resistance or virulence genes of importance have been identified (Shridhar et al., 2022). In poultry, supplementation of *E. faecium* 669 increased performance and immune response in a dose-response manner (Wu et al., 2019), whereas data on *E. faecium* 669 supplementation on performance of calves are lacking. During the preweaning period, dairy calves are also exposed to a variety of stressors that significantly affect their overall welfare, health, and performance (Hulbert and Moisá, 2016), including ones leading to enteric diseases (Angeli et al., 2020). Therefore, strategies that support gastrointestinal tract health, while improving pre- and postweaning performance of the herd, are warranted and should be investigated. Hence, we hypothesized that *E. faecium* 669 supplementation during the preweaning period would improve overall performance of dairy calves. Therefore, our objective was to evaluate the effects of *E. faecium* 669 supplementation during the preweaning period on performance of dairy calves (experiment 1), but also whether changing the dose of the probiotic during the preweaning period would affect this parameter (experiment 2). A secondary objective was to start assessing the potential benefits of such direct-fed microbials on diarrhea occurrence and severity in preweaning dairy calves.

Both experiments were conducted at the Institute of Animal Science of the Lithuanian Veterinary Academy (Baisogala, Lithuania). All animals utilized in both experiments were cared for in accordance with the practices outlined in the Guide for the Care and Use of Agricultural Animals in Agricultural Research and Training (FASS, 2010) and the experimental activities approved by the Institutional Animal Care and Use Committee (IACUC) from the Institute of Animal Science of Lithuanian Veterinary Academy.

In experiment 1, twenty 4-d-old Holstein calves (10 males and 10 females) were included herein (initial BW 41 kg ± 2.1 kg) for a 60-d experimental period. Calves were ranked by initial BW and sex and randomly assigned into 1 of 2 treatment groups: (1) no probiotic supplementation (n = 5 male and 5 female calves; **CON**) and (2) supplementation of *E. faecium* 669 during the preweaning period (n = 5 male and 5 female calves; LACTIFERM, 2.0 × 10^11^ cfu/g; Chr. Hansen A/S; **DFM**). During the entire experimental period, DFM was mixed into the whole milk at a rate of 2.0 × 10^10^ cfu/kg of whole milk. Regardless of the treatment group, all animals were housed individually and fed the same starter supplement (33.0% barley, 25.0% wheat, 16.0% soybean meal, 14.0% rapeseed cake, 8.0% fodder yeast, 2.7% dicalcium phosphate, 1.0% mineral-vitamin mix, and 0.3% sodium carbonate), medium-quality first-cut grass-legume sward hay (8.0% CP), grass silage, and water in amounts to ensure ad libitum intake, as well as 6 L of whole milk, containing (DFM) or not (CON) the probiotic, twice a day (3 L per feeding). Once a week, whole milk samples from CON and DFM were collected, pooled across weeks at the end of the study, and sent to the laboratory of the Lithuanian State Veterinary (Kaunas, Lithuania) for colony-forming unit counts of the probiotic.

Full individual BW measurements were taken on d 0, 20, 40, and 60 (at weaning) of the experimental period, and ADG was calculated. Throughout the experimental period, starter supplement intake was measured daily, and feed efficiency (**FE**) calculated.

In experiment 2, thirty 4-d-old Holstein calves (14 males and 16 females) were included (initial BW 40.0 kg ± 1.9 kg) for a 63-d experimental period. Calves were ranked by initial BW and sex and randomly assigned into the same treatment groups as described in experiment 1 (CON and DFM; n = 7 male and 8 female calves per group). The DFM supplementation period was further divided into periods I (from d 0 to 21) and II (from d 22 to 63), with weaning occurring when animals were 67 d of age. During the entire experimental period, DFM was mixed into the whole milk at a rate of 1.5 × 10^10^ and 2.5 × 10^9^ cfu/kg of whole milk per calf per day for periods I and II, respectively. Animal housing, starter supplement, whole milk sampling for determination of colony-forming units, and whole feeding management were the same as in experiment 1, with the exception that grass silage was not fed in experiment 2. Full individual BW measurements were taken on d 0, 21, 42, and 63 (at weaning) of the experimental period, and ADG was calculated from d 0 to 21, d 21 to 42, and d 42 to 63. Throughout the experimental period, starter supplement intake was measured daily and FE (gain-to-feed ratio) calculated during the aforementioned periods.

All occurrences and diseases that required any type of pharmacological intervention were registered routinely. The health status of the calves was monitored daily with particular attention given to the occurrence of diarrhea, using the following stool consistency score: 0 = firm, no signs of diarrhea, 1 = soft, slightly loose fecal consistency, and 2 = liquid, very loose fecal consistency. The personnel offering the treatments and providing the health score were not the same, minimizing any potential influence or bias on the assessment and resulting score.

Performance variables (ADG and FE) were considered the main variables of interest for both trials and power calculation demonstrated that 10 experimental units per treatment would be the lowest sample size to detect 100 g/head per day of difference on ADG, whereas 110 g of feed/gain would be adequate for FE. For both experiments, all data were analyzed using calf as the experimental unit and the SAS software (version 9.4, SAS Institute Inc.). Performance data were analyzed using the MIXED procedure of SAS, using initial BW as a covariate, as repeated statements with calf(treatment × sex) used as subject, and the covariance structure tested included compound symmetry and first-order regressive, whereas the later was chosen as it gave the lowest Akaike information criterion. All results were reported as least squares means. Fixed effects included treatment, day/period, and sex, whereas calf was the only random effect. For diarrhea occurrence (score ≥1), GLIMMIX procedure of SAS was used, considering calf as the random variable, and treatment and week as fixed effects. For all the data, significance was set at *P* ≤ 0.05 and tendencies were denoted if *P* > 0.05 and *P* ≤ 0.10. Moreover, results are reported according to the main effects if no interactions were significant.

In experiment 1, all milk samples analyzed for *E. faecium* 669 counts had values within the expectation (data not shown). No treatment × sex interactions were observed for any of the variables analyzed herein (*P* ≥ 0.48) and no calves died during the study. The covariate was not significant for BW, starter and starter + hay intake, and gain-to-feed ratio (*P* ≥ 0.19), but did affect ADG (*P* = 0.04). A treatment × day interaction was observed for BW of the calves (Table 1), as DFM-supplemented calves were heavier (*P* ≤ 0.02) on d 40 and 60 of the study, finishing the experiment 6.5 kg heavier than the CON calves (7.8% improvement at weaning; Table 1). Moreover, inclusion of DFM in whole milk improved ADG (*P* < 0.01), without further effects on gain-to-feed ratio (*P* = 0.18; Table 1).

**Table 1:** Performance of dairy calves supplemented or not (CON; n = 10) with *Enterococcus faecium* 669 (DFM; n = 10) during the preweaning period in experiment 1^1^

Item	Treatment		SEM	*P*-value		
	CON	DFM		Treatment	Time	Interaction
BW, kg			1.30	0.02	<0.0001	0.02
d 20	48.6	50.8				
d 40	64.7^b^	69.2^a^				
d 60	83.5^b^	90.0^a^				
ADG, kg	0.705	0.823	0.0263	<0.01	<0.0001	0.95
Solid feed^2^						
Starter intake, kg/d	0.515	0.508	0.0243	0.89	<0.001	0.21
Starter + hay intake, kg/d	0.644	0.631	0.0284	0.82	<0.001	0.27
Gain to feed, kg/kg	1.10	1.28	0.237	0.18	<0.001	0.44

a,b Within a row, different letters indicate differences at the *P* < 0.05 level.

1 The *Enterococcus faecium* 669 (LACTIFERM, Chr. Hansen A/S) was mixed in whole milk that was offered daily at a rate of 6 L per calf per day. The daily dose of *E. faecium* 669 was 2.0 × 10^10^ cfu/kg of whole milk.

2 Calculation based on the amount of starter supplement and hay consumed by the calves.

In agreement with experiment 1, all *E. faecium* 669 counts were within the expectation (data not shown) in the whole milk of experiment 2. Moreover, no calves died herein and no treatment × sex interactions were observed for any of the variables analyzed (*P* ≥ 0.22). The covariate was significant for BW, ADG, starter intake, starter + hay intake, and gain-to-feed ratio (*P* ≤ 0.04). Treatment × day and treatment × week interactions were observed for BW and gain-to-feed ratio (*P* ≤ 0.05), whereas a tendency was observed for ADG (*P* = 0.06; Table 2). Dairy calves supplemented with DFM were 1.8 and 3.5 kg heavier on d 42 and at weaning versus CON (+ 3.1% and 4.7%, respectively; *P* ≤ 0.03) and had a greater ADG from d 21 to 42 and 42 to 63 (*P* ≤ 0.01). Moreover, mean starter intake tended to be greater (*P* = 0.07) and total (starter + hay) intake and gain-to-feed ratio was greater for DFM-supplemented calves versus CON from d 42 to 63 (*P* ≤ 0.04; Table 2). Nonetheless, 95.2% and 89.5% of diarrhea cases were observed in the first 4 wk of the experiment for CON and DFM calves, respectively; thus, diarrhea data were analyzed from wk 1 through 4 of the experiment. Diarrhea occurrence tended to be reduced in DFM-supplemented versus CON calves (*P* = 0.08; 40.0% vs. 20.0%, respectively; SEM = 8.43), whereas weekly diarrhea occurrences were 50%, 30%, and 10%, respectively (week effect; *P* < 0.001; SEM = 8.1).

**Table 2:** Performance of dairy calves supplemented or not (CON; n = 15) with *Enterococcus faecium* 669 (DFM; n = 15) during the preweaning period in experiment 2^1^

Item	Treatment		SEM	*P*-value		
	CON	DFM		Treatment	Time	Interaction
BW, kg			0.58	0.01	<0.0001	<0.001
d 21	47.0	47.7				
d 42	58.8^b^	60.6^a^				
d 63	74.8^b^	78.3^a^				
ADG, kg			0.0145	<0.01	<0.0001	0.06
d 0–21	0.347	0.380				
d 21–42	0.563^b^	0.615^a^				
d 42–63	0.763^b^	0.840^a^				
Starter intake, kg/d	0.306	0.391	0.0243	0.07	<0.0001	0.11
Starter + hay intake, kg/d	1.14	1.24	0.029	0.08	<0.0001	0.11
Gain-to-feed ratio, kg/kg			0.019	0.13	<0.0001	0.01
d 0–21	0.48	0.46				
d 21–42	0.55	0.57				
d 42–63	0.70^b^	0.76^a^				

a,b Within a row, different letters indicate differences at the *P* < 0.05 level.

1 The *Enterococcus faecium* 669 (LACTIFERM, Chr. Hansen A/S) was mixed in whole milk that was offered daily at a rate of 6 L per calf per day. The daily dose of *E. faecium* 669 was 1.5 × 10^10^ and 2.5 × 10^9^ cfu/kg of whole milk from d 0 to 21 and d 21 to 63, respectively.

Probiotics must be alive to bring the expected benefits to the host (Markowiak and Śliżewska, 2018) and *E. faecium* is one of the strains that has been used as a probiotic source in poultry, swine, and ruminants, such as dairy cows (Nocek and Kautz, 2006) and beef cattle (Dias et al., 2022), but to the best of our knowledge, data on the effects of *E. faecium* 669 on performance of dairy calves are lacking. Supplementation of *E. faecium* 669 as a probiotic source to preweaning dairy calves improved performance of the herd, an effect that became more apparent as the study progressed. In other words, the differences on BW (experiments 1 and 2) and ADG (experiment 2) between CON and DFM calves increased as the preweaning phase advanced. Previous studies in the literature demonstrated that preweaning performance directly affects the productive performance of a lactating mature cow (Soberon et al., 2012; Bach et al., 2021). In experiment 2, the occurrence of diarrhea episodes in the first 4 wk of the trial decreased when DFM was supplemented, suggesting that acute diarrhea is the primary issue in the first 4 wk of a calf's life (Wang et al., 2022) and that the gut of the calves was more resilient in tolerating potential pathogenic challenges. Lactic acid bacteria, such as *E. faecium*, inhibit the growth of pathogenic bacteria by decreasing the pH in the large intestine and through competitive attachment (Riddell et al., 2010). Moreover, *E. faecium* facilitates systemic and intestinal local mucosal immune responses (Siepert et al., 2014), increases the absorptive and secretory capacity of jejunal mucosa, improves intestinal barrier function, enhances disease resistance to pathogenic infection, prevents or treats diarrhea, and increases growth performance in pigs (Wang et al., 2014). In pigs, administration of *E. faecium* 669 enhanced gut health by promoting the growth of beneficial bacteria and inhibiting the proliferation of gut pathogens (Pajarillo et al., 2015).

In preweaning calves, diarrhea is one of the main challenges faced by dairy producers, as its occurrence may range from 20% to 37% (NAHMS, 2012; Angeli et al., 2020), supporting our results from experiment 2. Heinrichs and Heinrichs (2011) demonstrated that the number of days a calf is sick before 4 mo of age influences the entire productive performance of the lactating dairy cow, highlighting the importance of this specific period for the profitability of the dairy operation. Moreover, as current public concerns regarding the appearance and spread of antibiotic-resistant bacteria keep growing, novel and natural technologies are warranted to benefit the health and performance of preweaning dairy calves. Among these technologies, natural substances that alleviate the stress of the herd (Angeli et al., 2020), prebiotics (Magalhães et al., 2008), and probiotics (Renaud et al., 2019; Wang et al., 2022) have been suggested as feasible alternatives to reduce the occurrence and duration of enteric diseases, as well as the cost of pharmacological intervention.

It is noteworthy that in experiment 2, reducing the DFM dose by 6 times and offering a similar dose of *E. faecium* 669 as experiment 1 after 3 wk on trial did not compromise the performance and health of the calves. In fact, benefits on performance of the calves did not occur until d 21 of experiment 2, when most of the diarrhea cases have also been observed, leading us to hypothesize that feeding *E. faecium* 669 might have (1) prevented pathogen colonization of the digestive tract (Fuller, 1989), (2) allowed an early establishment and colonization of beneficial bacteria in the gut of the calves (Wang et al., 2022), (3) ensured the balance of good microbiota, even in the presence of a pathogenic challenge condition (Signorini et al., 2012), or (4) induced a greater nutrient utilization that improved overall health and performance of preweaning calves (or a combination of these). Therefore, feeding *E. faecium* 669 at different doses during the preweaning period, the most critical window of potential pathogen contamination, was able to support the health of the calves and improve performance during the preweaning period. The daily and steady supply of *E. faecium* 669 during the preweaning period, at a standard or greater dose, positively supported the health of the animals at a small scale. However, additional research efforts should focus on evaluating *E. faecium* 669 under a more commercial and large-scale setting to confirm such benefits. Moreover, there is an opportunity to evaluate the interaction and response of standard doses of *E. faecium* 669 under an extreme challenge for diarrhea occurrence.

In summary, supplementation of a direct-fed microbial containing *E. faecium* 669 to preweaning dairy calves improved herd performance, even when different doses of the DFM were provided during preweaning. Moreover, diarrhea occurrence tended to be reduced by DFM supplementation, warranting additional efforts to understand such effects in a large-scale setting and the underlying modes of action and how the microbiota of preweaning calves is altered following *E. faecium* 669 supplementation.
